# Preliminary Biological Evaluation of Novel ^99m^Tc-Cys-Annexin A5 as a Apoptosis Imaging Agent

**DOI:** 10.3390/molecules18066908

**Published:** 2013-06-10

**Authors:** Chunxiong Lu, Quanfu Jiang, Minjin Hu, Cheng Tan, Yu Ji, Huixin Yu, Zichun Hua

**Affiliations:** 1 Key Laboratory of Nuclear Medicine, Ministry of Health & Jiangsu Key Laboratory of Molecular Nuclear Medicine, Jiangsu Institute of Nuclear Medicine, Wuxi 214063, China; E-Mails: luchunxiong@jsinm.org (C.L.); jiangquanfu@163.com (Q.J.); dmrlc@163.com (C.T.); 2 Jiangsu Target Pharma Laboratories Inc., Changzhou High-Tech Research Institute of Nanjing University, Changzhou 213164, China; 3 The State Key Laboratory of Pharmaceutical Biotechnology, Nanjing University, Nanjing 210093, China

**Keywords:** cys-annexin A5, ^99m^Tc-labelled, biodistribution, apoptosis imaging

## Abstract

A novel annexin A5 derivative (cys-annexin A5) with a single cysteine residue at its C-terminal has been developed and successfully labeled in high labeling yield with ^99m^Tc by a ligand exchange reaction. Like the 1st generation ^99m^Tc-HYNIC-annexin A5, the novel ^99m^Tc-cys-annexin A5 derivative shows in normal mice mainly renal and, to a lesser extent, hepatobiliary excretion. In rat models of hepatic apoptosis there was 283% increase in hepatic uptake of ^99m^Tc-cys-annexin A5 as compared to normal mice. The results indicate that the novel ^99m^Tc-cys-annexin A5 is a potential apoptosis imaging agent.

## 1. Introduction

Apoptosis or programmed cell death (PCD) plays an important role not only in physiology but also in pathology [1,2]. Therefore, the detection and quantification of apoptosis *in vivo* are of significant clinical value for diagnosis and assessment of therapeutic efficacy.

One of the hallmarks of cells going into apoptosis is the externalization of the phospholipid phosphatidylserine (PS) at the cell membrane [3,4]. Annexin A5, a 36-kDa human protein, shows Ca^2+^-dependent binding to negatively charged phospholipid surfaces and was discovered as a vascular anticoagulant protein [5,6]. The anticoagulant activity is based on the high-affinity for PS. These characteristics make a derivative of annexin A5 a suitable candidate for imaging of apoptosis.

Several annexin A5 conjugates tagged with fluorophores and bifunctional chelators (BFC) have been shown to retain the PS affinity of native annexin A5 [7,8,9,10,11,12,13]. *In vivo* detection, identification, and characterization of apoptosis is even more difficult. There has only been limited success so far in monitoring apoptosis by standard, noninvasive *in vivo* imaging modalities such as radionuclide imaging methods. The radionuclide of choice for the labeling of annexin A5 for single-photon imaging applications is often considered to be technetium-99m (^99m^Tc). Besides favorable photon energy and decay properties, it is continuously available at a reasonable cost from a generator. Several ^99m^Tc-labeling chelators have been reported, and they include *n*-1-imino-4-mercaptobutyl (imino) [14], ethylenedicysteine (EC) [10,15], 4,5-bis(thioacetamido)pentanoyl (BTAP) [7,16,17], mercaptoacetyltriglycine (MAG-3) [8] and hydrazinonicotinamido (HYNIC) [18,19,20]. One of the most widely used derivatives has been the ^99m^Tc-HYNIC-annexin A5 for imaging apoptosis by single photon emmission computed tomography (SPECT) [11,12,13]. Conjugation of HYNIC to annexin A5 for labeling with technetium-99m is usually done by targeting an amino group of one of the 21 lysine residues using *N*-succinimidyl HYNIC, but this method is rather non-specific as any of the –NH_2_ groups could be targeted.

Recent studies reveal that after structural modification in the recombinant expression annexin A5 can be directly marked with ^99m^Tc, such as ^99m^Tc(CO)3-HIS-cys-AnxV [21], ^99m^Tc-annexin-V-117 [22] and ^99m^Tc-His10-annexin V [23]. New annexin A5 molecules labeled by site-specific methods will greatly improve sensitivity for detecting cell death *in vivo* [24]. Recently, Professor Hua Zichun and his colleagues have developed a novel annexin A5 derivative (cys-annexin A5) with a single cysteine residue at C-terminal [25]. Their findings show that the the detection signal of fluorescein isothiocyanate (FITC)-cys-annexin A5 is greater than that of FITC-wild-type annexin A5.

We herein report the labeling and preliminary *in vivo* evaluation of the novel site-specific ^99m^Tc-cys-annexin A5 in normal mice and in rat models of apoptosis induced by cycloheximide. In mice tracer uptake was studied by *ex vivo* biodistribution experiments and the results were compared to those of the 1st-generation ^99m^Tc-HYNIC-annexin A5. In a rat model of hepatic apoptosis, tracer uptake was studied by SPECT. Apoptosis was confirmed *in situ* on liver slices using the terminal deoxynucleotidyl transferase (TdT) dUTP nick endlabeling (TUNEL) assay.

## 2. Results and Discussion

### 2.1. Radiolabeling

Annexin A5 has been labelled with a number of isotopes including^99m^Tc, ^124^I, ^18^F and ^68^Ga [18,26,27,28,29]. Positron emission tomography (PET) with its higher sensitivity, better spatial resolution and the ability to better quantify the radiopharmaceutical uptake is superior to SPECT imaging, and the low uptake of ^18^F-annexin A5 in the liver, spleen and kidney might represent an advantage over ^99m^Tc-annexin A5 [30], however production of ^18^F-annexin A5 depends on the availability of an expensive on-site cyclotron for the production of fluorine-18 (t1/2 = 109.8 min). ^68^Ga has suitable physical properties for PET imaging. Recently, it was reported that ^68^Ga-annexin A5 has a similar biodistribution in mice compared with other ^99m^Tc-annexin A5 [29]. In the present study, site-specific labeling of cys-annexin A5 with ^99m^Tc by a ligand exchange reaction. In the presence of disodium edetate, ^99m^Tc-cys-annexin A5 was labeled with Na^99m^TcO_4_ by reduction with stannous chloride. 

**Figure 1:**
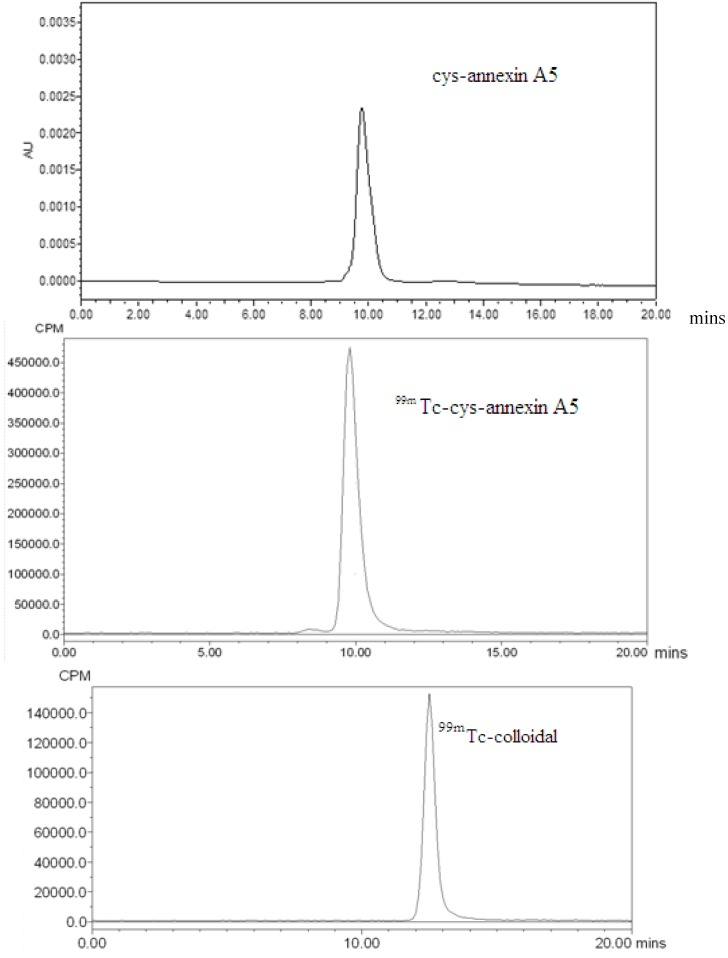

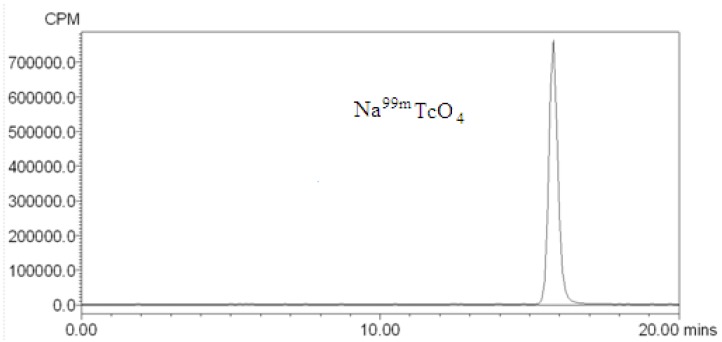
HPLC chromatograms (cys-annexin A5 t_R_ = 9.8 min (UV), ^99m^Tc-cys-annexin A5 t_R_ = 9.8 min, ^99m^Tc-colloidal t_R_ = 12.5 min and Na^99m^TcO_4_ t_R_ = 15.9 min).

HPLC analysis revealed cys-annexin A5 and ^99m^Tc-cys-annexin A5 that were eluted at a same retention time of 9.8 m, whereas ^99m^Tc-colloidal and the formation of free technetium (Na^99m^TcO_4_) eluted at a retention times of 12.5 m and 15.9 m, respectively (Figure 1). For each radiolabeled complex, the single peak in the HPLC-chromatogram clearly shows the formation of only one complex and excludes the possibility of residual Na^99m^TcO_4_ or other components.

According the HPLC analysis, the radiochemical purity of ^99m^Tc-cys-annexin A5 was greater than 95%. The radiolabeled compounds were used immediately after the formulation for both *in vitro* and *in vivo* studies.

### 2.2. Blood Kinetics Studies

**Figure 2:**
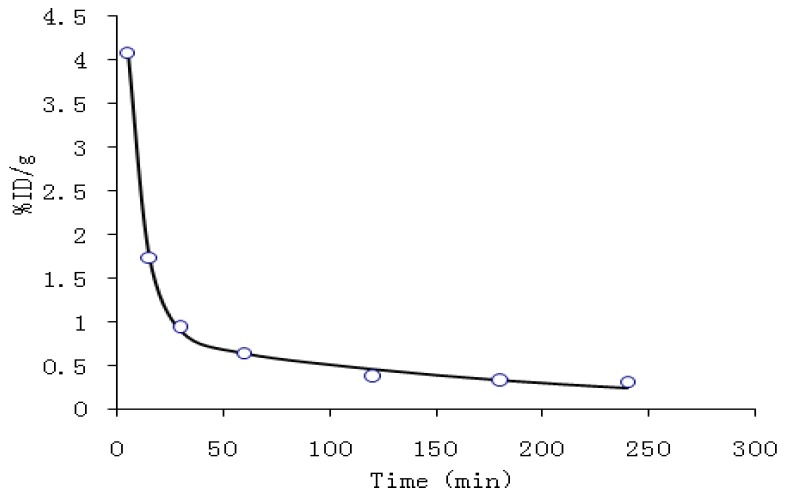
Pharmacokinetic curve in the mice for ^99m^Tc-cys-annexin A5.

Pharmacokinetic parameters were listed in Table 1. Figure 2 shows the blood clearance of ^99m^Tc-cys-annexin A5 in the mice 4 h post injection. Pharmacokinetics of ^99m^Tc-cys-annexin A5 comply with the two-compartment model with the pharmacokinetic equation of C = 5.972e^−0.123t^ + 0.877e^−0.005t^. The values of CL and AUC were 0.084 and 238, respectively.

**Table 1:** Pharmacokinetic parameters of the ^99m^Tc-cys-annexin A5 in mice.

Parameters	^99m^Tc-cys-annexin v
K_12_ (min^−1^)	0.079
K_21_ (min^−1^)	0.02
K_e_ (min^−1^)	0.029
CL (%ID/g/min)	0.084
T_1/2α_ (min)	5.619
T_1/2β_ (min)	129.465
AUC (%ID/g*min)	238.123

In the early phase, the blood clearance of ^99m^Tc-cys-annexin A5 was fast. After 1 h, the radioactivity concentration of the tracer agent in blood reaches an equilibrium which coincides with the pharmacokinetic parameters CL, AUC and the pharmacokinetic curves.

### 2.3. Biodistribution Studies

**Figure 3:**
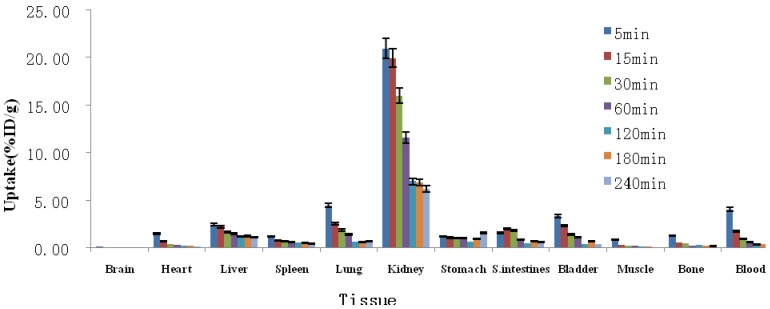
Biodistribution of ^99m^Tc-cys-annexin A5 in mice (mean ± SD, n = 5, %ID/g).

Biodistribution of ^99m^Tc-cys-annexin A5 was determined in ICR mice, and the data are shown in Figure 3 as the percentage administered activity (injected dose) per gram of tissue (%ID/g). Inspecting Figure 3, one can observe that ^99m^Tc-cys-annexin A5 was mainly accumulated in the kidney, which means that the clearance of ^99m^Tc-cys-annexin A5 was mainly through the renal pathway. There was moderate uptake of ^99m^Tc-cys-annexin A5 in the bladder, lung, and liver; uptake was low in the heart, spleen, stomach, bowels and skeletal tissue; uptake was very low in the brain and muscles. Most ^99m^Tc-labeling annexin A5 derivatives had a similar biodistribution *in vivo*, accumulated in the kidney and liver. Because of their different chelators, there were some differences in biodistribution between them. ^99m^Tc-imino-annexin A5, ^99m^Tc-EC-annexin A5, ^99m^Tc-BTAP-annexin A5 and ^99m^Tc-MAG3-annexin A5 showed higher uptake than ^99m^Tc-HYNIC-annexin A5 in intestines [7,8,10,14,16]. ^99m^Tc-HYNIC-annexin A5 had higher uptake than ^99m^Tc-MAG3-annexin A5 in kidney and liver [8]. ^99m^Tc-BTAP-annexin A5 was excreted significantly faster than ^99m^Tc-imino-annexin A5 [16]. Compared with ^99m^Tc-imino-annexin A5 [14,16], ^99m^Tc-EC-annexin A5 [15], ^99m^Tc-BTAP-annexin A5 [16] and ^99m^Tc-MAG3-annexin A5 [8], the degree of uptake of ^99m^Tc-cys-annexin A5 was relatively less in the intestine, and ^99m^Tc-cys-annexin A5 was suitable for the imaging of the abdomen.

Table 2 shows the biodistribution results in normal mice of novel ^99m^Tc-cys-annexin A5 and 1st-generation ^99m^Tc-HYNIC-annexin A5 at 60 min pi, expressed as %ID/g. The uptakes of ^99m^Tc-HYNIC-annexin A5 in spleen and kidney were 6.52 ± 1.02%ID/g and 114 ± 17.1%ID/g, respectively. However, the uptakes of ^99m^Tc-cys-annexin A5 in spleen and kidney were 0.60 ± 0.03%ID/g and 11.61 ± 1.12%ID/g, respectively, which were smaller than those of ^99m^Tc-HYNIC-annexin A5. Similar values of the uptakes of ^99m^Tc-HYNIC-annexin A5 and ^99m^Tc-cys-annexin A5 in stomach and small intestines. ^99m^Tc-HYNIC-annexin A5 has been successfully used in many studies, however it has certain limitations most notably, its very high uptake in the kidney – which results in high radiantion dose and may limit imaging in the abdomen [18]. Therefore ^99m^Tc-cys-annexin A5 maybe more suitable than ^99m^Tc-HYNIC-annexin A5 for imaging in the abdomen.

**Table 2:** Biodistribution of ^99m^Tc-HYNIC-Annexin A5 and ^99m^Tc-cys-annexin A5.

Tissue	%ID/g(60min)	
	^99m^Tc-cys-annexin A5	^99m^Tc-HYNIC-annexin A5 [7]
Heart	0.26 ± 0.03	1.20 ± 0.25
Liver	1.50 ± 0.15	6.65 ± 1.02
Spleen	0.60 ± 0.03	6.52 ± 1.56
Lung	1.42 ± 0.30	2.56 ± 0.68
Kidney	11.61 ± 1.12	114 ± 17.1
Stomach	1.07 ± 0.44	1.33 ± 0.19
S. intestines	0.89 ± 0.16	0.97 ± 0.09
Muscle	0.24 ± 0.14	0.37 ± 0.08
Bone	0.25 ± 0.03	0.78 ± 0.14
Blood	0.64 ± 0.15	1.77 ± 0.31

### 2.4. Imaging of Rat Model of Apoptosis

**Figure 4:**
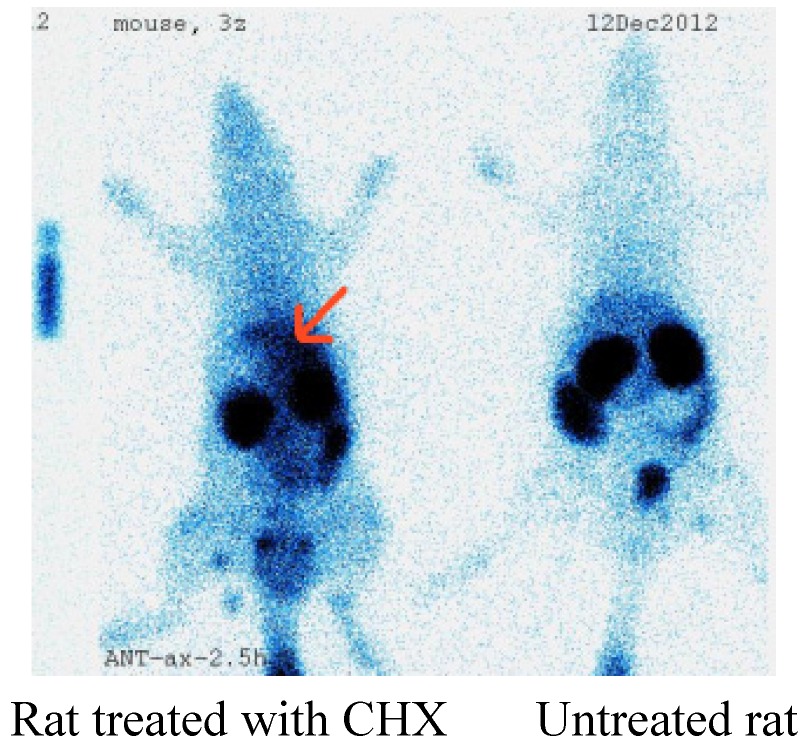
Planar imaging of rat model of apoptosis.

There were three rats treatment with cycloheximide to induce liver apoptosis and two rats as the control group. Figure 4 shows the planar images of normal and cycloheximide (CHX)-treated rats. ^99m^Tc-cys- annexin A5 tracer uptake in the liver was increased with CHX treatment (indicated by arrows). Biodistribution of ^99m^Tc-cys-annexin A5 in treated rats indicated that liver, spleen and kidney uptakes were 0.28 ± 0.01%ID/g,1.22 ± 0.03%ID/g and 5.21 ± 0.02%ID/g, respectively, at 190 m p.i. The uptakes in liver, spleen and kidney of control rats were 0.10 ± 0.005%ID/g, 0.25 ± 0.01%ID/g and 5.50 ± 0.03%ID/g, respectively, at 190 m p.i. The uptake ratios (treated/control) of liver, spleen and kidney were 2.83, 4.94 and 0.95, respectively, at 190 m p.i. There were no differences in the blood pool activity between treated and control rats. The changes observed in ^99m^Tc-cys-annexin A5 biodistribution as measured via SPECT correlated with the increase in cell death observed using TUNEL histochemistry. 

**Figure 5:**
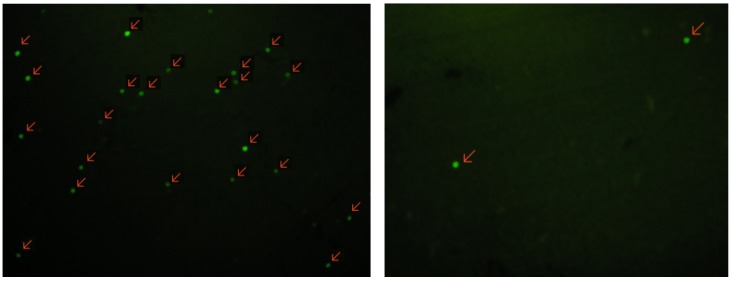
TUNEL staining of liver sections.

Analysis of TUNEL-stained sections confirmed that a clear increase in the number of TUNEL-positive cells was present in liver, 6 h following an i.p. injection of CHX (See Figure 5). Photomicrographs of the corresponding 5 µm liver section after TUNEL staining of CHX-treated rat (1st column) and control (2nd column) 6 h after CHX treatment. TUNEL-positive nuclei (green nuclei, indicated by arrows) are diffused in treated, but little in control mice and indicate apoptosis.

Rat treated with CHX    Untreated rat liver

### 2.5. Toxicity Test

The toxicity test was evaluated by the death and 48 h survival of the mice, which were injected with 0.5 mL ^99m^Tc-cys-annexin A5 (3.7MBq), respectively. Saline-injected (of the same volume) mouse group was used as the control group. As expected, the mice showed no signs of toxicity through the overall study period.

In summary, ^99m^Tc-cys-annexin A5 is a promising imaging agent in the detection of apoptosis and early assessment of tumor therapeutic efficacy. We plan to develop a lyophilized kit of cys-annexin A5 for ^99m^Tc-labeling for clinical use.

## 3. Experimental

### 3.1. General

All analytical chemical reagents employed were purchased from commercial sources and used without further purification. cys-Annexin A5 was supplied by Jiangsu Target Pharma Laboratories Inc. (Changzhou, China). Na^99m^TcO_4_ was supplied by Jiangsu Institute of Nuclear Medicine (Wuxi, China) HPLC was performed on a Waters 600-type high-performance liquid chromatography (Milford, MA, USA) equipped with a dural λ absorbance detector (Waters 2487), binary HPLC pump (Waters 1525) and Cd(Te) detector equipped with a scintillation analyzer (Perkin Elmer, Waltham, MA, USA). A Packard-multi-prias γ counter (made in USA), a fluorescence microscope (Olympas X51, Tokyo, Japan) and a Philips SKY Light single photon emission computed tomography instrument (SPECT) (San Francisco, CA, USA) were used. The animal experiments in this study were approved by the Animal Care and Ethnics Committee of Jiangsu Institute of Nuclear Medicine.

### 3.2. Radiochemical Synthesis of ^99m^Tc-cys-Annexin A5

In the labeling of ^99m^Tc-cys-annexin A5, cys-annexin A5 (50 µg) was dissolved in 50 µL PBS (0.1 mol/L, pH =7.4) in an evacuated nitrogen filled vial. To this solution, disodium edetate (1.5 mg) dissoleved in PBS (75 µL, 0.1 mol/L, pH =7.4), stannous chloride (20 µg) dissolved in HCl (20 µL, 0.1 mol/L) were added respectively. After addition of all reagents, [Tc-99m]pertechnetate solution (~18.5 MBq, 0.1 mL) was added into the vial. Reaction mixture volume used was about 0.3 mL. The vial was incubated at 37 °C in a water bath for 15 min.

### 3.3. Quality Control of ^99m^Tc-cys-Annexin A5

The radiochemical purity (RCP) and radiolabeling yield (RLY) of ^999m^Tc-cys-annexin v was determined by HPLC. The RCP of ^99m^Tc-cys-annexin A5 was determined a Waters 600-type high-performance liquid chromatography. The sample was passed through a millipore filter carefully and injected into the HPLC column (TSK-GEL swG2000SWXL, 300 × 7.8 mm 5 µm, Tosoh Bioscience Co., Ltd, Shanghai, China). The absorbance was measured on the UV detector at 278nm. Radioanalysis of the labeled compound was conducted using a Cd (Te) detector. The flow rate was adjusted to 0.8 mL/min and the isocratic mobile phase was 0.05 mol/L phosphate buffer (Ph = 7.0).

### 3.4. Biodistribution in Normal Mice of ^99m^Tc-cys-Annexin A5

Thirty-five ICR mice were randomly divided into seven groups and injected via the tail vein with ^99m^Tc-cys-annexin A5 in the volume of 0.2 mL and activity of approximately 3.7 MBq. Groups of mice were sacrificed at 5, 15, 30, 60, 120, 180 and 240 min after injection. The organs of interest (brain, heart, liver, lung, kidney, spleen and muscle etc.) were dissected and weighed, as well as 100 μL blood were taken from carotid artery. The activity for each sample was determined by a γ counter. Distribution of the radioactivity in different tissues and organs was calculated and expressed as percentage of injection dose per gram (%ID/g).

### 3.5. Animal Model of Apoptosis

Three male SD rats (288 ± 1g) were treated IV with 5 mg/kg cycloheximide to induce liver apoptosis. Two male SD rats (289 g and 291 g) were treated IV with saline as the control group. 3 h after the treatment, rats were injected via the tail vein with 0.2 mL (18.5 MBq) ^99m^Tc-cys- annexin A5 and 3 h later imaged with SPECT for 10 min. The organs of interest (liver, spleen and kidney) were dissected and weighed at the end of the scan. Liver samples were divided into 2 parts. Then, using aliquots of the liver, formalin-fixed paraffin-embedded specimens were prepared for Terminal deoxynucleotidyl Transferase dUTP nick end labeling (TUNEL) staining.

### 3.6. TUNEL Staining

Because our imaging studies were designed to determine the uptake and biodistribution of ^99m^Tc-cys-annexin A5 after chemically induced apoptosis, it was important to confirm apoptosis in the livers of treated rats by independent methods that provide quantitative results. A marker of apoptosis was scored by performing a TUNEL assay that measures DNA fragmentation, a characteristic feature of apoptosis. Terminal deoxynucleotide transferase adds labeled nucleotides to the 3′ termini at double-stranded breaks in the fragmented DNA. TUNEL assays were performed according to the manufacturer’s instructions, using the fluorescein-conjugated Colorimetric TUNEL Apoptosis Assay Kit (Beyotime Institute of Biotechnology, Shanghai, China). Briefly, slices were freed of paraffin through xylene and graded EtOH washes and then incubated with proteinase K (Beyotime Institute of Biotechnology) (2 mg/mL in 10 mmol/L Tris, pH 8.0). After proteinase digestion, the slides were equilibrated in pH 7.4 buffer, the terminal deoxynucleotide transferase enzyme and Biotin-dUTP labeling mix (Beyotime Institute of Biotechnology) were added, and the slides were incubated at 37 °C for 1 h in a humid chamber. The number of TUNEL-positive cells was counted on 10 randomly selected ×100 fields for each section by use of a Olympus fluorescence microscope.

## 4. Conclusions

Cys-annexin A5, a novel annexin A5 derivative with a single cysteine residue at the C-terminal, could be labeled with ^99m^Tc in high yields and high radiochemical purity. In normal mice, ^99m^Tc-cys-annexin A5 was rapidly cleared from the blood and excreted mainly through the renal pathway. Hepatic uptake of ^99m^Tc–cys-annexin A5 was significantly increased in the rats treated with CHX compared to controls, which correlated well with the increase in cell death observed using TUNEL histochemistry. These results indicate that the novel ^99m^Tc–cys-annexin A5 is a potential apoptosis imaging agent and further optimization of the cys-annexin A5 kit is nevertheless desirable in order to improve the potential clinical usefulness of this new technique.
